# Diversity of Culturable Bacteria from Endemic Medicinal Plants of the Highlands of the Province of Parinacota, Chile

**DOI:** 10.3390/biology12070920

**Published:** 2023-06-27

**Authors:** Daniel Moraga, Katina Latorre, Patricio Muñoz-Torres, Steffany Cárdenas, Alan Jofré-Quispe, José López-Cepeda, Luis Bustos, Cristóbal Balada, María Fernanda Argaluza, Pablo González, Leda Guzmán

**Affiliations:** 1 Laboratorio de Fisiología, Departamento de Ciencias Biomédicas, Facultad de Medicina, Universidad de Tarapacá, Arica 1000000, Chile; dmoraga@academicos.uta.cl; 2 Laboratorio de Microbiología, Departamento de Tecnología Médica, Facultad de Ciencias de la Salud, Universidad de Tarapacá, Arica 1000000, Chile; 3 Laboratorio de Patología Vegetal y Bioproductos, Facultad de Ciencias Agronómicas, Universidad de Tarapacá, Arica 1000000, Chile; 4 Departamento de Ciencias Históricas y Geográficas, Universidad de Tarapacá, Arica 1000000, Chile; 5 Departamento de Biología, Facultad de Ciencias, Universidad de Tarapacá, Arica 1000000, Chile; 6 Subdepartamento de Gestión de Farmacia, Servicio de Salud Arica, Arica 1000871, Chile; 7 Laboratorio de Química Biológica, Instituto de Química, Facultad de Ciencias, Pontificia Universidad Católica de Valparaíso, Valparaíso 2340001, Chile

**Keywords:** endemic medicinal plants, rhizosphere bacteria, 16S rDNA sequence, antibiotic activity, bioprospecting, ethnomedicine

## Abstract

**Simple Summary:**

An increasing diversity of bacteria with medically relevant biological properties is being discovered in natural environments where extreme physical conditions can select for unique chemistry. This research has allowed us to discover, in the desert region of the Chilean highlands in the province of Parinacota (3681–5104 m high), bacterial strains that live in the roots of an Aymara medicinal plant with impressive biological activities, such as hydrolytic enzymes, plant-growth-promoting traits, and antibacterial and antifungal properties. These discoveries have great value for society and medicine and should be further investigated for the benefit of global public health.

**Abstract:**

Endemic medicinal plants that grow at altitudes in northern Chile have been traditionally used for therapeutic applications by Aymara doctors. Several studies have analyzed the biological properties of these plants for therapeutic purposes. The aim was to characterize at molecular and biochemical levels the bacteria that live in the rhizosphere and roots from endemic medicinal plants that grow between 3681–5104 m.a.s.l. in the province of Parinacota. Thirty-nine bacteria were isolated from nine medicinal plants under our laboratory conditions. These bacteria were characterized by Gram stain, hydrolase production, plant-growth promotion, anti-fungal and antibacterial activities, and 16S rDNA sequencing. A phylogenetic study revealed the presence of three major phyla, *Actinomycetota* (46.2%), *Bacillota* (43.6%), and *Pseudomonadota* (10.3%). The rhizobacteria strains associated with the Aymara medicinal plant exhibited several interesting biological activities, such as hydrolytic enzymes, plant-growth-promoting traits, and antibacterial and antifungal properties, indicating their potential for developing new bio-based products for agricultural or clinical applications. These results are promising and highlight the need to point toward the search for explanations of the bio-molecular basis of the therapeutic effects of medicinal plants.

## 1. Introduction

A rich diversity of bacteria is associated with animals and plants, and there is growing evidence that they are essential for life and the sustainability of ecosystems [1]. The soil microbiota contributes to the sustainability of ecosystems since these are the main agents of nutrient recovery by regulating the dynamics of soil organic matter [2,3]. In extreme environments such as the Atacama Desert in the north of Chile, extremophile microorganisms survive in oligotrophic conditions [4] where extreme abiotic factors such as dryness [5], high UV radiation [6], and severe day-night temperature fluctuations [7] are believed to select for diversity with unique chemistry [8]. Microbial communities in the soil of the Atacama Desert have been well studied because this region is considered the dry edge for life [9,10,11,12,13,14,15,16,17,18,19,20,21,22] and its similarities with the soil of Mars [23]. A paradigmatic study of these harsh conditions in the Atacama Desert was conducted by Maza et al., [18], where the bacterial diversity of soils from three sites in the Atacama Desert was analyzed, finding that the soils were dominated by Proteobacteria, Actinobacteria, and other phyla, such as Firmicutes and Bacteroidetes. The microbiota in these soils were influenced by the saline conditions. The study by Rated et al., [24], discovered in the hyper-arid environment of the Atacama Desert that the bacterium *Streptomyces leeuwenhoek* from phyla Actinobacteria produces the antibiotic chaxamycin in soil water. Chaxamycin shows antibacterial effects against multi-resistant *Staphylococcus aureus* (MRSA) and antitumoral effects [25]. This last discovery is of great interest to health and microbiological research, both because of the need for increasingly new antibiotics due to the multi-resistance of human pathogenic bacteria and the better understanding of the biological relationship between bacteria and their host. Recently, in the year 2020, a study on the rhizospheres associated with two medicinal plants, *Baccharis scandens* and *Solanum chilense,* showed that the *Ascomycota* and *Basidiomycota* fungal phyla and *Actinobacteria* and *Proteobacteria* bacterial phyla were the dominant taxa related to both rhizospheres, but with differences in relative abundance at the family level [17]. Almost all the studies reported on microbial diversity in the Atacama Desert were carried out in the I and II Regions of Northern Chile. However, only two bacterial diversity studies have been reported in the same period in the province of Parinacota, in the XV region, the northernmost region of Chile, on the border with Peru. These few studies include the work of Dorador et al., [19], where the bacterial communities associated with water and sediments of the Chungará Lake, the Parinacota wetland, and the Piacota Lake were characterized using culture-independent techniques. Authors found the presence of several bacterial phyla, including members of *Bacteroidetes*, α-*Proteobacteria*, β-*Proteobacteria*, γ-*Proteobacteria*, and *Firmicutes*, among others. Meanwhile, Muñoz et al., [20] studied the biodiversity of plant-associated bacteria in five crops cultivated in high altitudes, revealing the presence of members of *Pseudomonas*, *Bacillus*, *Pantoea,* and *Erwinia*, among other genera. These bacteria were functionally characterized for plant-growth-promoting activities, showing the potential of these microorganisms for the development of new agricultural bioproducts. However, to date, there is no reported evidence of bacteria living in the rhizosphere of the roots of medicinal plants endemic to the province of Parinacota in the XV region of Chile. For this reason, it is possible to consider that this is the first study carried out to search for medicinal bioactive compounds from these bacteria in the northernmost region of Chile. It is reasonable to expect uniqueness in the metabolic and chemical capacities of these bacteria from the desert highlands, which allow them to survive in extreme conditions of ultraviolet radiation, dryness, temperature, and geographical altitude between 2300 and 5500 m.a.s.l. [8].

This study aimed to isolate and characterize bacteria with antibacterial properties from the rhizosphere and Aymara endemic plant roots in the province of Parinacota of the Chilean highlands. Bacterial identification and their taxonomic classification were carried out through the sequencing of the 16S ribosomal DNA (rDNA) region [26,27], resulting in the partial 16S gene amplicons encompassing hypervariable regions, which were sequenced and their taxonomic identification based on bioinformatics alignments against sequence databases was performed [28]. Phylogenetic analysis showed the presence of three prominent phyla, *Actinomycetota*, *Bacillota*, and *Pseudomonadota*. These bacteria exhibited promising biological activities that could allow the development of new bioproducts for clinical and agricultural purposes. Interestingly, among these bacteria, *Bacillus* sp. K64 from the medicinal plant *Baccharis boliviensis* showed antibacterial activity against the multi-drug-resistant S. aureus. These results are promising and highlight the need to focus on the search for further explanations based on the therapeutic effects of medicinal plants.

## 2. Materials and Methods

### 2.1. General

Trypticase soy agar (TSA), tryptic soy broth (TSB), brain heart infusion broth (BHI), Mueller-Hinton broth (MHB), Mueller–Hinton agar (MHA), and potato dextrose agar (PDA) were purchased from Oxoid (ThermoFisher Scientific, Waltham, MA, USA). Amoxicillin, Kanamycin, and Methicillin were purchased from Sigma-Aldrich (St. Louis, MO, USA). Clindamycin was acquired from Cayman (Chemical Company, Ann Arbor, MI, USA), and the McFarland standard turbidity was acquired from bioMérieux.

**Figure 1:**
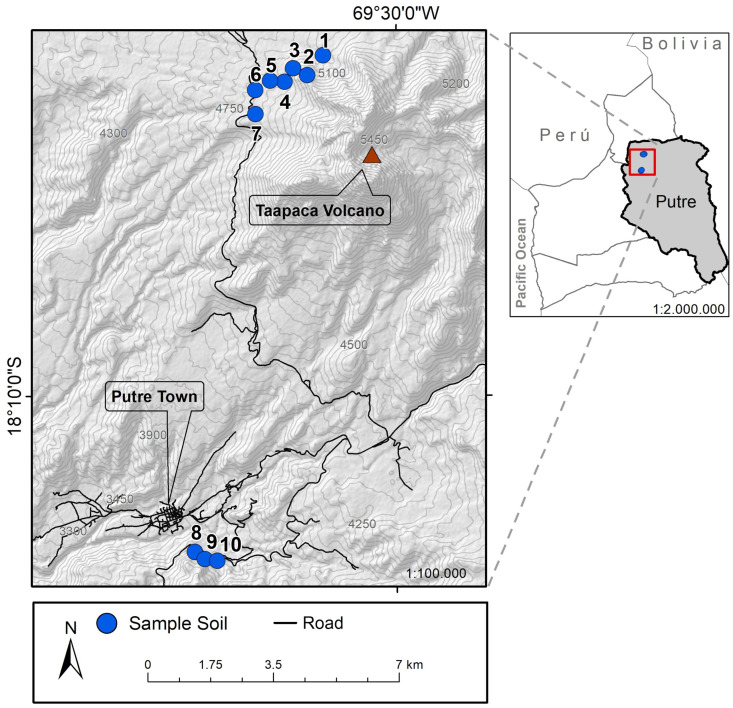
Putre and Taapaca volcano map. Sampling sites are indicated as enumerated blue points on the map.

Nine endemic medicinal plants were collected and extracted from the province of Parinacota, located in the extreme north of Chile, at 18° Lat S (Table 1). The geographic location of each sampling site is shown in Figure 1. These plants grow on the margins of the most desertic area on the planet: the Atacama Desert, specifically in the indigenous Aymara community of Putre, province of Parinacota, XV region. This ecosystem belongs to a large Andean orographic unit known as *Altiplano* (3200–6000 m.a.s.l.), whose vegetative floor results from its latitudinal position and unique high-mountain climatic distinctive qualities of the continent. These conditions allow for the generation of endemic and medicinal plants. 

Dr. Teófilo Cañari, Yatire of the Aymara culture in the locality of Putre, selected the study sites and collected the plant material. Collected samples were deposited in the herbarium of the Universidad de Tarapacá [29]. 

### 2.2. Bacterial Isolation

Bacteria were isolated from the rhizospheric soil and roots of nine medicinal plants growing under the conditions described above (Table 1 and Figure 1). Each root was extracted in a radius of 30 cm at a depth of 10 cm. A root sample was cut with sterile scissors, extracted with sterile forceps, and stored in a 15 mL Falcon tube. The corresponding soil sample was extracted with a sterile shovel near 30 cm of the root and stored in a 15 mL Falcon tube. These samples were transported to the Microbiology Specialties Laboratory of the Universidad de Tarapacá in a Coleman cooler container with refrigerated units. A physiological saline solution (15 mL) was added to each Falcon tube, vortexed for 30 s, and then 4 serial dilutions were made 1/10 in physiological saline. Each dilution was shaken for 30 s, plated on TSA, and incubated in an oven at 20 and 35 °C for 24 to 48 h. The morphological characteristics of 39 isolated colonies, such as size, color, and morphology, were evaluated and Gram stained. The isolates were transferred to a TSB or BHI liquid medium and then plated on solid medium plates. All the isolated bacteria were preserved in 15% glycerol at −80 °C until carrying out the tests for the presence of hydrolytic enzymes, the plant-growth-promotion test, and the growth-inhibition test of phytopathogenic fungi and bacteria associated with human infections.

### 2.3. Genetic Bacterial Identification

#### 2.3.1. Genomic DNA Extraction

The isolated bacterial strains were grown in TSB, and 3 mL were used for DNA extraction. DNA extraction was performed using the DNeasy UltraClean Microbial kit (QIAGEN, Germantown, MD, USA), according to the manufacturer’s instructions. The DNA integrity was analyzed using a 1.0% agarose gel, stained with ethidium bromide; the quantity and quality of the DNA were evaluated by measuring the absorbance to 260 nm and the ratio 260/280 nm using an EPOCH microplate reader (BioTek-Instruments, Winooski, VT, USA). 

#### 2.3.2. Sequencing Genomic DNA 

The 16S rDNA region was amplified by PCR using the 27F (5′-AGAGTTTGATCCTGGCTCAG-3′) and 1492R (5′-CTACGGCTACCTTGTTACGA-3′) primers. PCR was repeated using primers F984 (5′-AACGCGAAGAACCTTAC-3′) and R1378 (5′-CGGTGTGTGTACAAGGCCCGGAACG-3′) to verify the sequencing of one strain (K64). The PCR reaction was performed in a 20 μL reaction system on a VeritiTM 96-well Thermal Cycler (Thermo Fisher Scientific, Waltham, MA, USA), containing 2.5 mM MgCl_2_, 0.2 mM dNTPs, 0.25 mM of each primer, 50 ng of genomic DNA, and 0.5 mL of the enzyme (1 U/mL, KAPA HiFi HotStart, Biosystems). The temperature cycling parameters were performed as described by Muñoz et al. [20]. The PCR products (~1500 bp) were separated using 1.0% agarose gel stained with 1× Gel Red (Biotium, San Francisco, LA, USA), and the size of the amplifications was estimated using a 1 kb DNA Ladder (New England, BioLabs, Ipswich, MA, USA). PCR products were sequenced by external services (Macrogen, Seoul, Republic of Korea). The obtained sequences were manually edited using the ChromasPro software (http://technelysium.com.au/wp/chromaspro/ accessed on 15 November 2022) to remove low-quality bases. Forward and reverse sequences were assembled using the Megamerger tool (http://www.bioinformatics.nl/cgi-bin/emboss/megamerger accessed on 15 November 2022). The partial sequence was compared to GenBank using BLAST software employing the rRNA/ITS (16S ribosomal RNA sequences) database, excluding models, uncultured, and environmental sample sequences [30]. The partial 16S rDNA gene sequences were deposited in the GenBank nucleotide sequences databank under the accession numbers OQ225692-OQ225727 (Table S1).

#### 2.3.3. Phylogenetic Analysis

A phylogenetic tree was constructed using the 16S rDNA partial sequences of isolated bacteria obtained from the rhizosphere and roots of studied endemic plants. The partial sequence of isolated bacteria and selected sequences belonging to closely related bacteria retrieved from GenBank (closest hits obtained by Blast) were aligned using the ClustalW-software [31]. Phylogenetic relationships were carried out using the MEGA7 software [32] and considering the *Aquifex pyrophilus* strain Kol5a^T^ (GenBank accession number M83548) as the outgroup (selected by its high phylogenetic divergence). The phylogenetic tree was inferred from the multiple sequence alignments using the maximum likelihood method and a bootstrap analysis of 500 replicates to determine the reliabilities of each node [33].

### 2.4. Bacterial Characterization

#### 2.4.1. Gram Staining

Gram staining was carried out on a slide and observed under an Olympus light microscope (Oxford Instruments, Abingdon, England). Based on the coloration of the cell wall stain, the bacteria were classified as gram-positive or gram-negative [34].

#### 2.4.2. In Vitro Detection of Hydrolytic Activities

The hydrolytic activity of isolated bacteria was evaluated using four specific culture media and incubated from 24 to 48 h [35]. The protease, lipase, pectinase, cellulase, and amylase enzymes were determined on an agar plate containing a specific substrate for each enzyme. The protease activity was evaluated by growth on a skim milk agar medium, where a clear halo around the colony in a white background indicates the positive activity of casein degradation [36]. Lipase activity was detected by the method described by Slifkin [37], where the appearance of a white halo around the microorganism indicates the hydrolysis of Tween 80, producing fatty acids that react with the calcium cations present in the solid medium, forming a white precipitate; this white halo is indicative of a positive result. Cellulolytic activity detection was carried out in agar plates containing 1.0 g/L carboxymethylcellulose, 1.0 g/L peptone, 0.3 g/L urea, 2.0 g/L KH_2_PO_4_, 1.0 g/L (NH_4_)_2_SO_4_, 0.3 g/L CaCl_2_, 0.3 g/L MgSO_4_, 0.014 g/L ZnSO_4_, 0.002 g/L CoCl_2_, 0.05 g/L FeSO_4_, 0.016 g/L MnSO_4_, and 15.0 g/L agar. Cultures were incubated at room temperature for 5 days. After incubation, the plates were flooded with 0.1% Congo red solution for 20 min and then washed for 20 min with 1 M NaCl. Cellulase activity was observed as a transparent halo around the bacteria on a red background. Finally, the amylase activity was performed on agar plates containing 2.0 g/L starch, 3.0 g/L beef extract, 6.0 g/L NaCl, and 12.0 g/L agar. The inoculated plates were incubated at room temperature for one week and then flooded with a lugol solution for 20 min. Amylase activity was observed as a clear halo around the microorganism in a dark background [35,36,37]. All preparations and measurements were conducted in triplicate. 

#### 2.4.3. In Vitro Plant-Growth-Promoting Traits

Indole-3-acetic acid (IAA) production was determined by the colorimetric Salkowski’s method [38]. After centrifugation, the bacterial supernatant was recovered, and the cellular pellet was mixed with Salkowski’s reagent (0.5 M FeCl_3_ in 35% HClO_4_) in the ratio 1:2 (supernatant: Salkowski’s reagent). A color change from yellow to red was indicative of IAA production. The siderophores production was executed using the Chrome Azurol S (CAS) method described by Schwynand and Neilands [39], in which 500 μL of bacterial supernatant was mixed with 500 μL of CAS solution. A color change from blue to orange indicated the production of siderophores. The solubilization of inorganic phosphate was performed using the Pikovskaya (PVK) solid medium [40], where the PKV medium contained 10.0 g/L glucose, 0.5 g/L yeast extract, 0.5 g/L (NH_4_)_2_SO_4_, 0.1 g/L MgSO_4_·7H_2_O, 5.0 g/L Ca_3_(PO_4_)_2_, 0.2 g/L KCl, 0.002 g/L MnSO_4_·2H_2_O, 0.002 g/L FeSO_4_·7H_2_O, and 15.0 g/L agar. The results were determined by the appearance of a clearing zone around bacterial colonies after incubation was indicative of phosphate solubilization. Each bacterial isolate was detected using the NFb semisolid medium to evaluate the ability to fix nitrogen [41]. The NFb medium contained 5.0 g/L DL-malic acid, 0.5 g/L K_2_HPO_4_, 0.2 g/L MgSO_4_·7H_2_O, 0.1 g/L NaCl, 0.02 g/L CaCl_2_·2H_2_O, 0.5 g/L agar, 2 mL (per liter) micronutrient solution, 2 mL (per liter) bromothymol blue solution (0.5% in 0.2 N KOH): 4 mL (per liter) Fe (III)-EDTA (1.64% *w*/*v*), and 1 mL (per liter) vitamin solution. pH was adjusted to 6.8 using NaOH. The micronutrient solution contained 0.4 g/L CuSO_4_·5H_2_O, 0.12 g/L ZnSO_4_·7H_2_O, 1.4 g/L H_3_BO_3_, 1.0 g/L Na_2_MoO_4_·2H_2_O, and 1.5 g/L MnSO_4_·H_2_O. The vitamin solution consisted of 100 mg/L of biotin and 200 mg/L of pyridoxal HCl. Nitrogen fixation was determined by developing a sub-superficial whitish ‘veil-like’ pellicle after incubation. All preparations and measurements were conducted in triplicate. 

#### 2.4.4. In Vitro Growth Inhibition of Phytopathogenic Fungi

An antagonism assay was carried out according to the procedure described by Sepúlveda-Chavera [42] using a plate of 94 mm with 20 mL of PDA for all tests. Agar disks from a one-week-old actively growing colony were used to inoculate fresh PDA plates with the phytopathogenic fungi *Botrytis cinerea*, *Monilinia fructicola*, *Phytium* sp., *Geotrichum candidum*, or *Fusarium oxysporum* (isolates Bot1, Mon1, Phy1, Geo1, and Fus1, respectively; Microbial Culture Collection of the Universidad de Tarapacá) in the center of the plate, and 20 μL of each bacteria isolate (1 × 10^6^ UFC/mL) were inoculated in 5 mm-wells at 2.5 cm from the center of the plate. Plates only inoculated with fungi in the center were used as a control. Cultures were incubated at room temperature for 5–7 days, and the presence of a halo around the wells indicated the fungal inhibition growth. All preparations and measurements were performed in triplicate. 

#### 2.4.5. In Vitro Growth Inhibition of Pathogenic Bacteria

The Kirby–Bauer Disk diffusion method and Clinical and Laboratory Standards Institute (CLSI) guidelines [43] were performed as screening to evaluate antibacterial activity from bacterial isolates. *Staphylococcus aureus* ATCC25923 *Escherichia coli* ATCC 25922, and *Klebsiella pneumoniae* ATCC 1705 were used as reference strains. All the antibacterial tests were carried out using fresh colonies and suspended in 0.85% NaCl to a turbidity of 0.5 McFarland (1.5 × 10^8^ CFU/mL). Then, each bacteria suspension was spread with a sterile cotton swab on plates of 94 mm with a fixed quantity of MHA to *S. aureus*, *E. coli,* and *K. pneumoniae*. The antibacterial activity was also evaluated on Methicillin-resistant *S. aureus* (MRSA) and Clindamycin-resistant (PUCV Doc 1505 and 1507) strains donated by Dr. Levican in the Microbiology Laboratory from Medical Technology School, Pontificia Universidad Católica de Valparaiso. Each bacterial suspension (20 μL) was added to a 5 mm sterile filter paper disc and dried at room temperature. Then, the paper discs were gently placed in the center of each plate. The antibiotic disks Amoxicillin (20 µg), Kanamycin (30 µg), Methicillin (10 µg), and Clindamycin (2 µg) were used as the control. Then, the plates were incubated aerobically at 37 °C overnight for 24 to 48 h, and inhibition zone diameter (IZD) was measured using a ruler. All preparations and measurements were conducted in triplicate. 

#### 2.4.6. Antimicrobial Activity of Supernatants from Bacteria

A bacterial suspension was prepared from bacteria that presented an inhibitory capability on pathogenic bacteria. Briefly, a bacterial suspension in 5 mL of TSB and BHI was grown at 30 °C for 24 h under shaker agitation at 200 rpm. The bacterial suspension was centrifuged at 10,000× *g* for 5 min for bacterial cell sedimentation, and the supernatant obtained was sterile-filtered through 0.2-micron filters. Then, 20 μL of cell-free supernatants were added to a 5 mm sterile filter paper circle, dried, and placed on pathogenic bacteria in the center of each plate as described above. 

## 3. Results

### 3.1. Bacterial Isolation, Identification, Distribution, and Functional Characterization

Plant-associated bacteria were isolated from nine endemic medicinal plants, as indicated in Table 1, from Parinacota Province in Chile in an altitudinal range of 3681–5104 m.a.s.l. A total of 39 bacterial isolates were obtained from their rhizosphere and roots, 16 isolates were extracted from the roots of the plants, and 22 isolates were extracted from the rhizosphere. Interestingly, from the total of the isolated bacteria, 26 isolates were found in the higher sample site (4840–5104 m.a.s.l.), while 13 isolates were located at the lower sample site (3681–3683 m.a.s.l.) as is shown in Figure 1. According to their morphology, 38 were bacilli, of which 16 were gram-positive, 23 were gram-negative, and the rest presented characteristics of cocci. Additionally, most colonies were rough, smooth-edged, slightly convex, and of many sizes, some even forming satellite colonies; some were yellow, white, brown, and red. In general form, all bacterial colonies were visible after 24–48 h of incubation to 28 or 30 °C on plates of nutritive medium supplemented with agar. 

The sequences of 39 bacterial isolates (Table S1) were compared to the 16S rDNA bacterial sequences available in GenBank (Table S2) to identify the microorganisms found in the plants and the rhizosphere and to analyze their phylogenetic relationship. The results show that isolates belong to the *Bacillus* genus (10 isolates), followed by *Pseudarthrobacter* (5 isolates), *Paenarthrobacter* (4 isolates), *Microbacterium* (4 isolates), and *Priestia* (3 isolates) genera. One or two members of the isolates of *Stenotrophomonas*, *Advenella*, *Staphylococcus*, *Micrococcus*, *Rhodococcus*, *Leifsonia*, *Pseudomonas*, *Ureibacillus*, *Cytobacillus*, *Arthrobacter,* and *Candidimonas* genera were obtained. The additional amplification of the strain K64 fusion F984-R1378 (to confirm the identity of this isolate) indicated that strains presented a 99.74% and 99.49% of homology with *Bacillus pumilus* and *B. safensis*, respectively (GenBank accession number: MN181289.1; MW850559.1).

**Figure 2:**
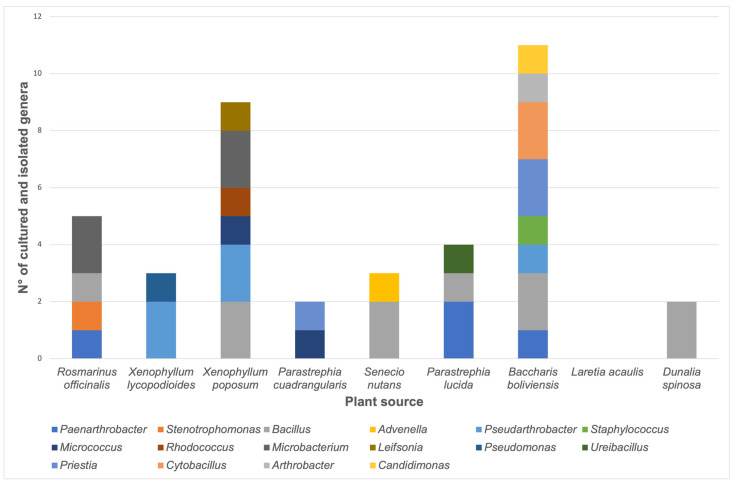
Number of stable bacterial cultures according to the plant source. The quantity of each genus is represented as a color bar, as indicated in the image.

The number of isolates belonging to each genus according to the plant source is shown in Figure 2. It was possible to isolate four bacteria from *P. lucida* plants, including members of *Paenarthrobacter*, *Bacillus,* and *Ureibacillus* genera. The bacterial genera obtained from *R. officinalis* samples include two members of the *Microbacterium* genus and one isolate of *Paenarthrobacter*, *Stenotrophomonas,* and *Bacillus* genera. For *S. nutans* samples, two *Bacillus* and one *Advenella* were isolated. For *X. lycopodioides*, two *Pseudarthrobacter* and one *Pseudomonas* were obtained. Eleven isolates were obtained from *B. boliviensis* samples, including members of *Paenarthrobacter*, *Bacillus*, *Pseudarthrobacter*, *Staphylococcus*, *Priestia*, *Cytobacillus*, *Arthrobacter,* and *Candidimonas* genera. Nine isolates from *X. poposum* plant samples included members of *Bacillus*, *Pseudarthrobacter*, *Micrococcus*, *Rhodococcus*, *Microbacterium*, and *Leifsonia* genera. For the *D. spinosa* sample, two *Bacillus* were isolated; meanwhile, one *Micrococcus* and one *Priestia* were obtained from the *P. cuadrangularis* plant. However, it was not possible to isolate any bacterium from *Laretia acaulis*.

All isolated bacteria were characterized to explore the production of hydrolytic enzymes, their plant-growth-promoting traits, and their ability to inhibit phytopathogenic fungi (Table 2 and Table S1). Protease and amylase were the most abundant detected hydrolytic activity, with 28 and 22 isolates showing a positive reaction, respectively. Meanwhile, lipase and cellulase activities were detected in 9 and 13 isolates, respectively. The ability to solubilize inorganic phosphate was the most abundant plant-growth-promoting trait detected in 26 isolates. Elemental nitrogen fixation (9 isolates) and the production of IAA (4 isolates) and siderophores (14 isolates) were also present in the isolated bacterial cultures. The strains *Paenarthrobacter* sp. K52 and *Microbacterium* sp. K57 showed biocontrol activity against *Fusarium oxysporum* and *Botrytis cinerea*, respectively; meanwhile, six isolates inhibited the growth of phytopathogenic fungi *Macrophomina phaseolina* and *Monilinia fructicola*.

**Figure 3:**
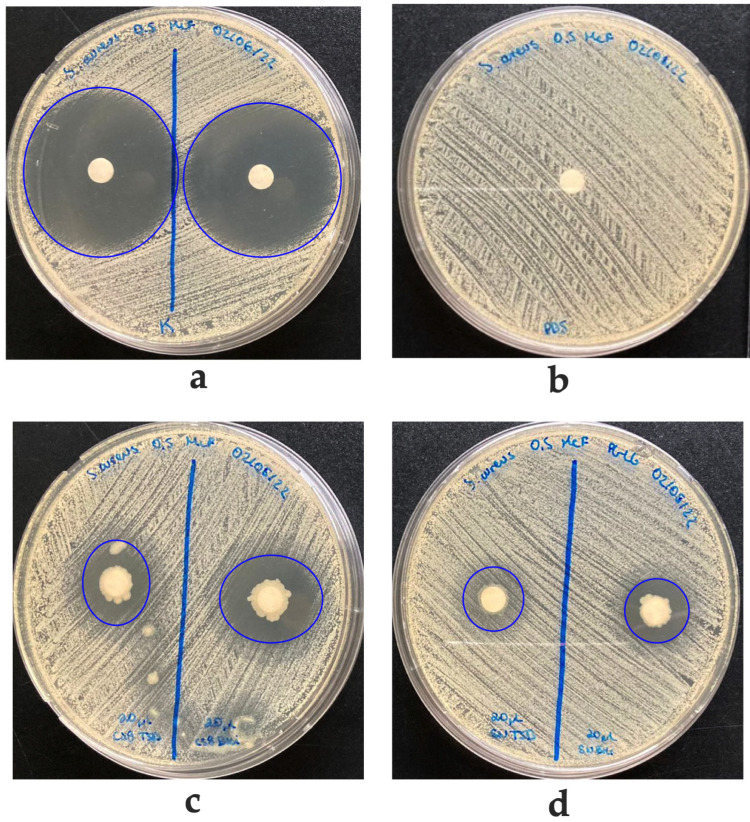
Agar diffusion antibacterial activity of K64 strain against *Staphylococcus aureus* ATCC 25923. (**a**) Inhibition halo using Kanamycin as a positive control. (**b**) PBS on filter paper as negative control (IZD of 162 mm). (**c**) Inhibition halo with 20 μL of K64 strain grown in TSB (left, IZD of 16.2 mm) and BHI (right, IZD of 21.3 mm). (**d**) Inhibition halo with supernatant of K64 growing in TSB (weak IZD of 15 mm) or BHI (IZD of 15.8 mm), respectively.

Interestingly, *Bacillus* sp. K64 showed an antibiotic power against *S. aureus* ATCC 25923, MSRA, and Clindamycin-resistant *S. aureus* (Figure S1), but it was negative against *E. coli* and *K. pneumoniae*. When this strain was grown in a TSB medium, an IZD of 16.2 mm was observed, while in a BHI medium, the IZD was 21.3 mm, as shown in Figure 3. In addition, antibacterial activity was found in the culture medium, as shown in Figure 3d, when the supernatant cell-free for strain K64 was used. The result has shown a visible halo (without bacteria) where the strain was grown in BHI with a size of 15.8 mm. However, an inhibition halo was not observed when the strain was grown in TSB, showing a weak growth around the paper disc instead. This strain with antibiotic power was found in *Baccharis boliviensis,* also known as Chaka-Tola plants (3683 m.a.s.l., point 8 Figure 1). 

### 3.2. Phylogenetic Analysis

**Figure 4:**
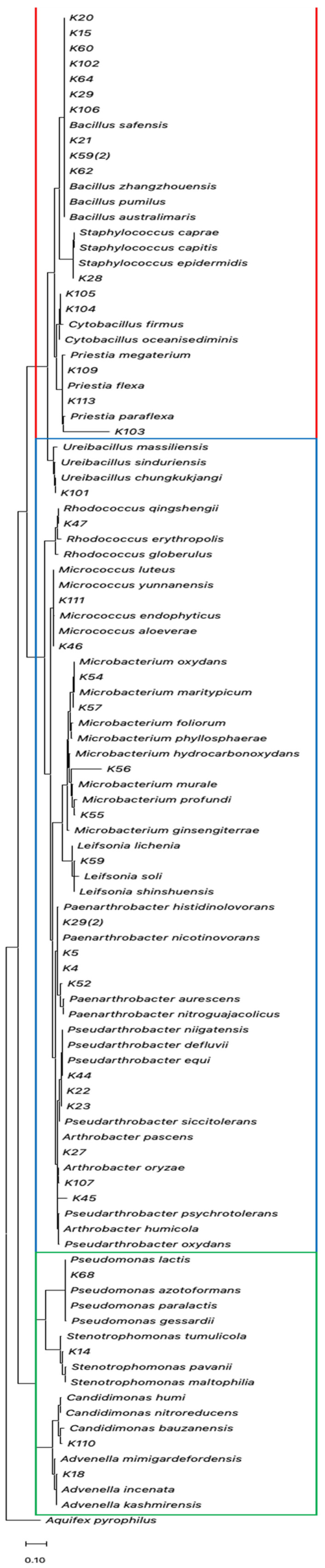
The phylogenetic tree is based on the 16S rDNA gene sequences of the isolated bacteria and their closely related species obtained from GenBank. The tree was constructed using the maximum likelihood method. The presence of *Actinomycetota*, *Pseudomonadota*, and *Bacillota* phyla are indicated as blue, green, and red boxes, respectively. The accession number of each retrieved sequence is indicated in Table S2.

Bacterial isolates were analyzed using a fragment of ~1000 bp (without ambiguous bases) of the 16S rDNA gene (Figure 4). The molecular characterization showed that the isolated bacteria were affiliated with three prominent phyla (Table S1): *Actinomycetota* (18 isolates, 46.2%), *Bacillota* (17 isolates, 43.6%), and *Pseudomonadota* (4 isolates, 10.3%). Four bacterial classes were obtained, *Actinomycetia* class being the most frequently isolated (18 isolates, 46.2%), followed by *Bacilli* (17 isolates, 43.6%), and γ-proteobacteria (2 isolates, 5.1%) and β−proteobacteria (2 isolates, 5.1%). Bacteria belonging to *Bacillus* were the most frequent genus isolated from medicinal plants from the province of Parinacota, reaching 25.6% (10 of 39) of the samples.

## 4. Discussion

In this study, 39 bacteria were isolated from the rhizosphere and the roots of nine plants that grow between 3681 and 5104 m.a.s.l in the province of Parinacota and are used by the Aymara people for their medicinal benefits (Table 1) [44]. The rhizosphere of plant roots consists of numerous dangerous and beneficial organisms, such as pathogenic fungi, oomycetes, bacteria, and nematodes. Soil microorganisms, among other functions, improve the availability and absorption of nutrients, and nitrogen fixation processes play a key role in plant-growth promotion under different environmental conditions [8,9,10,11,12,13,14,15,16,17,18,19,20,21,22,23,24,25,26,27,28,29,30,31,32,33,34,35,36,37,38,39,40,41,42,43,44,45]. In this context, the bacteria found in the rhizosphere and roots of *S. nutans* (Chachacoma), *P. cuadrangularis* (Siputula), *D. spinosa* (Yara), *B. boliviensis* (Chaka-Tola), *R. officinalis* (Sallika), *P. lucida* (Noa-Tola), *X. poposum* (Pura-Pura) and *X. lycopodioides* (Jakana) plants (Table 1) were studied at the physical, biochemical level through microbiological tests, identification by sequencing of the 16S rDNA gene, detection of hydrolytic enzymes, plant-growth-promoting traits, growth inhibition of phytopathogenic fungi, and antibiotic activity against nosocomial multi-resistant bacteria of clinical importance. 

Soil organic matter (SOM) is the material produced originally by plants or animals that is returned to the soil, goes through a decomposition process, and is an essential factor in determining soil quality and fertility. This process is mediated by extracellular enzymes, including extracellular microbial hydrolases that degrade macromolecules to soluble substrates for their assimilation [46]. According to Table 2 and Table S1, protease activity was the most frequent activity detected in isolated bacteria (71.8%) and was present in the 3 detected phyla. Moreover, starch degradation through amylase production was detected in 22 isolates (56.4%); cellulase activity was present in 13 isolates (33.3%); and lipid degradation was present in 9 isolates (23.1%). The hydrolytic activity was also detected in endophytic bacteria associated with plants of the Poaceae family in Turkey [47]. The authors demonstrated that bacteria produced lipases, proteases, amylases, cellulases, pectinases, and xylanases with relative frequencies of 74.2%, 65.6%, 55.4%, 32%, 21.8%, and 7.8%, respectively. Moreover, Jalgaonwala and Mahajan [48] analyzed the presence of hydrolytic enzymes in endophytic fungi and bacteria associated with seven medicinal plants in India. In this study, 14 bacteria and 24 fungi were isolated from the aerial and underground parts of the plants. The relative frequencies of protease, amylase, cellulase, and lipase activities were 76.3%, 55.3%, 26.3%, and 31.6%, respectively. Their results are consistent with our analysis, in which the frequency of these activities was similar, with proteolytic activity being the most frequently detected.

Bacterial communities associated with desert plants play pivotal roles, contributing to growth and stress tolerance, such as high exposure to solar radiation and suppression of diseases by phytopathogens, allowing plants to survive under extreme conditions [22]. Desert soils are characterized by high salinity, very low water retention, and low nutrient levels, which are the prevailing conditions in Parinacota province [20,49]. The mechanisms associated with plant-growth-promoting bacteria (PGPB) include the following: (i) production of indole-3-acetic acid; (ii) production of the enzyme ACC deaminase; (iii) solubilization of inorganic phosphates; (iv) nitrogen fixation; and (v) production of siderophores [50].

Among the plant-growth-promoting traits detected in plant-associated bacteria isolated from medicinal plants of the province of Parinacota, inorganic phosphate solubilization was the most frequently determined trait, of which 26 isolates tested positive. Meanwhile, elemental nitrogen fixation was less frequent (4 isolates). These results are not in agreement with the work of Muñoz et al. [20], who analyzed the plant-growth-promoting characteristics of bacteria associated with ancestral crops isolated from oregano, alfalfa, maize, potato, and grapevine samples from Belén, Codpa, Molinos, Poconchile and Socoroma localities in the Arica and Parinacota Region, Chile. These authors determined that phosphate solubilization was also the most frequently detected plant-growth-promoting trait (122 isolates of 180). Furthermore, IAA production was determined in 115 isolates, N_2_ fixation in 103 isolates, and siderophores production in 46 of 180 isolates. These differences could be attributed to the plant source and its particularly associated microbiome. Plants influence their microbiome due to their capacity to produce and release chemical signals into their environment [51]. Root exudates are associated with signaling in plant-microbe interaction, allowing the plant to establish a particular rhizosphere, which could be beneficial or detrimental to different species of microorganisms [51]. The selection of microorganisms from the soil is driven by the host, modulated by the production of salicylic acid and phenols released into the exudates, and the evolutionary history of the plant [52]. 

Furthermore, agricultural practices modify the soil’s physicochemical properties, influencing the microbial communities’ composition. For example, organic fertilizers increase the diversity and heterogeneity of soil microorganisms [53]. Considering the mentioned information, it is possible to think that the microbial composition obtained by Muñoz et al., [20] was more heterogeneous due to the agricultural management of the soils with animal manure. Meanwhile, the lower diversity of bacteria obtained in this work could be explained by the type of soil, extreme UV radiation and temperature changes, reduced organic matter, and symbiotic relationships with other organisms where wild medicinal plants grow [20]. Moreover, considering the nature of the plants in both studies, it could be possible to conclude that plant signaling differs between both systems. In this sense, microbiome selection would be distinct.

Plant-associated bacteria naturally inhabit the rhizosphere and increase plant growth and yield. PGPB, including members of the *Azospirillum*, *Rhizobium*, *Pseudomonas,* and *Bacillus* genera, enhance the growth of different types of crops [54]. Furthermore, they have attracted particular attention for their ability to enhance productivity, sustainability, and profitability when food security and rural livelihood are paramount priorities. These bacteria not only play an essential role in helping plants to establish and grow in nutrient-deficient conditions but also participate in the antagonism of diseases caused by phytopathogenic microorganisms. Biocontrol mechanisms involved in pathogen suppression by plant-associated bacteria include substrate competition for iron, nutrients, space, antibiotic production, and lytic enzyme production, among others [55]. Eight of thirty-nine isolates showed in vitro antagonistic activity against phytopathogenic fungi (Table 2 and Table S1), and seven of them were able to produce protease enzymes. Specific enzymes can degrade or lyse cell walls of phytopathogens through the production and secretion of lytic enzymes and indirectly help in plant growth and development, including proteases. This enzyme exhibits a promising potential due to its different applications in the industry. Currently, this enzyme has also been recognized for its outstanding applicability in the field of agriculture. 

Further work on biocontrol demonstrated the ability of lytic enzymes, including extracellular protease, to antagonize several plant pathogens [56]. For example, an antagonistic strain of *Bacillus amyloliquefaciens* isolated from the rhizosphere of *Corchorus olitorius* (Jute) showed protease-mediated biocontrol activity against *Macrophomina phaseolina, F. oxysporum*, *Fusarium semitectum*, and *Alternaria alternata* [57]. In this work, further studies are needed to demonstrate the mechanisms associated with the growth inhibition of phytopathogenic fungi for bacteria obtained from medicinal plants of the province of Parinacota.

The *Bacillus* sp. isolate K15, K21, and K64 showed positive lipase activity, a common activity in bacteria such as *Bacillus* sp., *Pseudomonas* sp., and *Streptomyces* sp., among other bacteria [58]. Recently, the lipase produced from the *Bacillus* species has attracted much attention because of its biotechnological potential in the food and pharmaceutical industry [59,60]. *Bacillus* lipases have been purified and biochemically characterized from *Bacillus* found in different soil as *B. pumilus* isolated from Antarctic soil or *B. pumilus* RK31 isolated from oil-contaminated soil samples [59,61,62]. A similarity of 97% was encountered in the 16S rDNA between the K64 strain and the RK31 strain reported sequence described by Kumar et al. [62]. Bacteria of the genus *Bacillus* as *B. subtillis*, *B. pumilus,* and *B safensis,* have been isolated from terrestrial ecosystems, extreme sites such as marine sediment, and contaminated areas [63]. Nevertheless, desert soil bacteria associated with medicinal plants have yet to be explored. Our study contributes to the knowledge of rhizospheric bacteria associated with medicinal plants that grow in the soil conditions where these bacteria were found—the high-altitude areas of the Atacama Desert. 

In San Pedro de Atacama, there is already the antecedent of a bacterium from the desert, *Streptomyces leeuwenhoek*, which can inhibit the growth of *S. aureus* through its active compound chaxamycin [24,25]. This discovery has been of great interest both for public health and for microbiological research, which motivates us to continue with our research, given that there is this precedent of a bacterium from the Atacama Desert in the Antofagasta region with antibiotic power that prestigious national and international research groups have studied. One of the highlights of this study is the result of antimicrobial activity in the K64 bacterial strain. This bacterium strain grows in the rhizosphere and roots of *B. boliviensis* (Chaka-Tola). It has shown the ability to inhibit the growth of *S. aureus* (ATCC 25923, MRSA, and Clindamycin-resistant) (Figure S1). Several studies have described that environmental stress conditions such as pH, oxygen levels, and soil structure also affect the microorganisms in the rhizosphere. The presence of sugars, amino acids, organic acids, and vitamins released by plant roots provides a nutrient flow, allowing for the growth of different organisms such as fungi and bacteria [51,52,64]. These rhizospheric microorganisms produce antimicrobial metabolites, including antibiotics, enzymes, and secondary metabolites such as phenolics and flavonoids compounds [65], which can inhibit the growth of other microorganisms in the soil, including plant pathogens, and help to protect plants from diseases [45]. 

Many rhizobacteria, such as *Streptomyces* spp. [24] and *Bacillus* spp., are known for producing metabolites bioactive with antimicrobial activity, such as peptides enzyme, volatile compounds, non-ribosomal peptides, and polyketides, among others [64,66,67]. In our case, we observed that the supernatant of the K64 strain exhibited an inhibition halo around paper discs on *S. aureus* ATCC25923 and two resistant *S. aureus* (MRSA and Clindamycin) in the agar diffusion antibacterial assays. These results suggest that these bacteria could release some metabolites that might be used to eliminate other bacteria found in the ecological site. Therefore, our preliminary results open a window for a genetic study and metabolism of this type of bacteria to find new molecules with antibiotic properties. 

Consequently, the Atacama Desert is an open-air laboratory that has sparked the scientific community’s interest. High-quality research can be generated from this desert to study extremophile microorganisms and their biotechnological applications [18,20,21,22,24,68]. It is also provocative to hypothesize that bacteria living in symbiosis with endemic medicinal plants may facilitate or contribute to their medicinal properties. Plant-associated bacteria produce a wide array of compounds that possess antimicrobial activity against phytopathogenic microorganisms. These compounds play a crucial role in safeguarding plants from microbial attacks. Several studies have been conducted on the bioactivity of medicinal-plant-associated microbiota, revealing numerous bioactive molecules with diverse functions, including characterized and novel antimicrobial compounds [69]. Jasim et al. [70] isolated a *Bacillus* strain from the traditional Indian medicinal plant *Bacopa monnieri* L. which possesses an inhibitory effect on the growth of the phytopathogenic fungi *Rhizoctonia* sp., *Sclerotium* sp., and *Phytophtora* sp. 

Furthermore, this bacterium exhibited antibacterial activity against pathogenic bacteria *E. coli*, *Salmonella enterica* Tiphy, *B. subtilis*, *S. aureus*, and *K. pneumoniae*. The study of Jasim et al. revealed the production of important compounds with antibiotic activity, including surfactin, iturin, and fengycin. Meanwhile, Roy et al. [71] isolated an endophytic *Bacillus* strain from the medicinal plant *Andrographis paniculata* Nees, which displayed broad-spectrum antimicrobial activity against bacterial pathogens such as *B. subtilis*, *B. cereus*, *Vibrio parahaemolyticus*, *Aeromonas caviae*, *Proteus vulgaris*, and *Pseudomonas aeruginosa*. Analysis of the individual extracts revealed the presence of three distinct metabolites, including an anthracene derivative. Moreover, Castillo et al. [72] identified a group of bioactive substances called munumbicins from *Streptomyces* sp. NRRL 30562, which was isolated from the medicinal plant *Kennedia nigriscans*. This group of substances exhibited activity against plant-pathogenic fungi and human-pathogenic bacteria, including antibiotic-resistant strains. In addition to these activities, several bacteria from the rhizosphere have been reported to possess antitumor activity as endophytic bacteria—*Lysinibacillus*, *Peribacillus*, and *Bacillus*—isolated from *A. sessiliflora*, where metabolites extracted from cultures of these bacteria with ethyl acetate showed growth inhibition of more than 90% against cervical adenocarcinoma, kidney adenocarcinoma, and lung carcinoma cells [73]. In the same line, Conti et al. [74] identified that metabolites from a strain of the genus *Streptomyces* associated with the Brazilian medicinal plant *Lychnophora ericoides* showed antitumoral activity against human cancer cell lines such as colon cancer, melanoma, and others.

These examples reveal that the bacteria that live in association with medicinal plants could facilitate the protection of human health and, thanks to them, contribute to the medicinal properties of these plants.

More research is needed to uncover the relationship between the root microbiome, its host-plant interaction, and its medicinal properties. Following the findings of this study, the associated researchers have initiated a study to characterize the biochemistry and antimicrobial properties found in strain K64.

## 5. Conclusions

Our research has revealed a unique microbiome associated with medicinal plants in the province of Parinacota, which possess several interesting properties that make them able to thrive in the desertic conditions of this region. Our analysis of the culturable bacteria in this microbiome revealed the prevalence of *Actinomycetota*, *Bacillota*, and *Pseudomonadota* phyla, with notable activities including hydrolytic enzyme production, plant-growth promotion, and inhibition of phytopathogenic fungi. These activities could conduct the development of new bioproducts useful for agriculture. Interestingly, we found the existence of a rhizobacterium of the *Bacillus* genus named K64, associated with the medicinal plant *Baccharis boliviensis* with antibiotic activity against multi-resistant *S. aureus.* The antibacterial activity is present in the bacterial growth medium, suggesting that it could be a bioactive metabolite release as a defense against other microorganisms that share the same habitat. Based on these results, it would be interesting to study the properties of *Bacillus* sp. isolate K64 and conduct the purification and characterization of bioactive metabolite in depth. Nowadays, our research groups are studying antibacterial activities and properties.

## Figures and Tables

**Table 1:** Name and Geospatial data for all sites sampled.

Site	Common Name	Scientific Name	Voucher N°	Latitude °S	Longitude °W	Altitude (m.a.s.l.)
1	Sallika	*Rosmarinus officinalis*	HRU1100	18.0810	69.5192	4888
2	Jakana	*Xenophyllum lycopodioides*	HRU1101	18.0859	69.5234	5104
3	Pura-Pura	*Xenophyllum poposum*	HRU1102	18.0842	69.5271	4951
4	Pura-Pura	*Xenophyllum poposum*	HRU1102	18.0876	69.5294	4867
5	Siputula	*Parastrephia cuadrangularis*	HRU1104	18.0873	69.5332	4807
6	Chachacoma	*Senecio nutans Sch. Bip*	HRU1105	18.0897	69.5371	4789
7	Noa-Tola	*Parastrephia lucida*	HRU1106	18.0957	69.5370	4830
8	Chaka-Tola	*Baccharis boliviensis*	HRU1107	18.2061	69.5533	3686
9	Llareta	*Laretia acaulis*	HRU1108	18.2079	69.5507	3683
10	Yara	*Dunalia spinosa*	HRU1109	18.2084	69.5475	3681

**Table 2:** Functional characterization of stable bacterial cultures from medicinal plants of the province of Parinacota.

Activity	N° Isolates with Positive Reaction	Relative Frequency (%)
Protease	28	71.8
Lipase	9	23.1
Cellulase	13	33.3
Amylase	22	56.4
Auxin production	4	10.3
Siderophores production	14	35.9
N_2_ fixation Phosphate solubilization	9	23.1
	26	66.7
*Botrytis cinerea* *	1	2.6
*Fusarium oxysporum* *	1	2.6
*Geotrichum candidum* *	0	0.0
*Phytium* sp. *	0	0.0
*Macrophomina phaseolina* *	6	15.4
*Monilinia fructicola* *	6	15.4

* Biocontrol activity against phytopathogenic fungus.

## Data Availability

Data is contained within the article or Supplementary Material.

## Supplementary Materials

The following supporting information can be downloaded at: https://www.mdpi.com/article/10.3390/biology12070920/s1. Table S1: Identification and biochemical characterization of bacteria isolated; Table S2: Accession number of retrieved sequences from GenBank for phylogenetic analysis. Figure S1: Agar diffusion antibacterial activity of K64 strain on antibiotic-resistant *S. aureus.* The sequences were deposited at GenBank with accession number OQ225692-OQ225727.
