# Supplementary material for: Diversity of Culturable Bacteria from Endemic Medicinal Plants of the Highlands of the Province of Parinacota, Chile
Source: Biology (Basel). 2023 Jun 27;12(7):920. doi: 10.3390/biology12070920 (PMC10376134; doi:10.3390/biology12070920)
Supplement: Supplementary file 1 [file biology-12-00920-s001.zip › Figure S1.pdf]

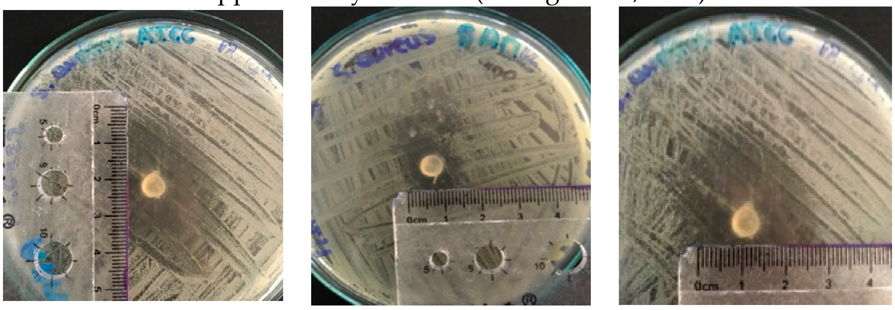

(**a**) (**b**) (**c**)

Figure S1. Agar diffusion antibacterial activity of K64 strain. The K64 strain was grown in BHI and 20 µL of supernatants cell free was used to test the antibacterial activity against antibiotic resistant *S. aureus*. (**a**) Inhibition halo observed on Clindamycin resistant *S. aureus* (IZD of 24,2 mm). (**b**) Inhibition halo observed on Methicillin resistant *S. aureus* (IZD of 12.4 mm). (**c**) Inhibition halo on *S. aureus* ATCC 25923 (IZD of 27 mm), used as a control of assay. The plates were incubated by 48 h.
