# Supplementary material for: Diversity of Culturable Bacteria from Endemic Medicinal Plants of the Highlands of the Province of Parinacota, Chile
Source: Biology (Basel). 2023 Jun 27;12(7):920. doi: 10.3390/biology12070920 (PMC10376134; doi:10.3390/biology12070920)
Supplement: Supplementary file 1 [file biology-12-00920-s001.zip › Table S2.pdf]

Table S2. Accession number of retrieved sequences from GenBank for phylogenetic analysis

Reference strain	GenBank accession number
Aquifex pyrophilus Kol5a	NR_029172.1
Leifsonia soli TG-S248	NR_116501.1
Leifsonia lichenia 2Sb	NR_112644.1
Leifsonia shinshuensis DB 102	NR_043663.1
Microbacterium hydrocarbonoxydans BNP48	NR_042263.1
Arthrobacter pascens DSM 20545	NR_026191.1
Arthrobacter humicola KV-653	NR_041546.1
Arthrobacter oryzae KV-651	NR_041545.1
Ureibacillus massiliensis 4400831	NR_043092.1
Ureibacillus sinduriensis BLB-1	NR_116552.1
Ureibacillus chungkukjangi 2RL3-2	NR_109669.1
Cytobacillus firmus NBRC 15306	NR_112635.1
Cytobacillus oceanisediminis H2	NR_117285.1
Rhodococcus globerulus DSM 43954	NR_026184.1
Rhodococcus erythropolis N11	NR_037024.1
Rhodococcus qingshengii CCM 4446	NR_145886.1
Priestia paraflexa RC2 16S	NR_135732.1
Priestia megaterium NBRC 15308	NR_112636.1
Priestia flexa NBRC 15715	NR_113800.1
Micrococcus endophyticus YIM 56238	NR_044365.1
Micrococcus aloeverae AE-6	NR_134088.1
Micrococcus luteus NCTC 2665	NR_075062.2
Micrococcus yunnanensis YIM 65004	NR_116578.1
Candidimonas humi SC-092	NR_117011.1
Candidimonas nitroreducens SC-089	NR_117010.1
Candidimonas bauzanensis BZ59	NR_108569.1
Microbacterium maritypicum DSM 12512	NR_042351.1
Microbacterium oxydans DSM 20578	NR_044931.1
Staphylococcus caprae ATCC 35538	NR_024665.1
Staphylococcus capitis MAW 8436	NR_027519.1
Staphylococcus epidermidis Fussel	NR_036904.1
Pseudarthrobacter oxydans DSM 20119	NR_026236.1
Pseudarthrobacter psychrotolerans YJ56	NR_174315.1
Pseudarthrobacter siccitolerans 4J27	NR_108849.1
Pseudarthrobacter niigatensis LC4	NR_041400.1
Pseudarthrobacter equi IMMIB L-1606	NR_117032.1
Pseudarthrobacter defluvii 4C1-a	NR_042573.1
Advenella incenata CCUG 45225	NR_042702.1
Advenella mimigardefordensis DPN7	NR_121716.1
Advenella kashmirensis PK1	NR_146816.1
Stenotrophomonas pavanii LMG 25348	NR_118008.1

Stenotrophomonas tumulicola T5916-2-1b	NR_148818.1
Stenotrophomonas maltophilia ATCC 13637	NR_112030.1
Paenarthrobacter aurescens DSM 20116	NR_026233.1
Paenarthrobacter nitroguajacolicus G2-1	NR_027199.1
Paenarthrobacter histidinolovorans DSM 20115	NR_026234.1
Paenarthrobacter nicotinovorans DSM 420	NR_026194.1
Pseudomonas gessardii CIP 105469	NR_024928.1
Pseudomonas paralactis DSM 29164	NR_156987.1
Pseudomonas azotoformans NBRC 12693	NR_113600.1
Pseudomonas lactis DSM 29167	NR_156986.1
Bacillus safensis NBRC 100820	NR_113945.1
Bacillus australimaris MCCC 1A05787	NR_148787.1
Bacillus zhangzhouensis MCCC 1A08372	NR_148786.1
Bacillus pumilus ATCC 7061	NR_043242.1
Microbacterium ginsengiterrae DCY37	NR_116483.1
Microbacterium foliorum P 333/02	NR_025368.1
Microbacterium murale 01-Gi-001	NR_117603.1
Microbacterium phyllosphaerae P 369/06	NR_025405.1
Microbacterium profundi Shh49	NR_044321.1
